# Molecular insights into *Cassava brown streak virus* susceptibility and resistance by profiling of the early host response

**DOI:** 10.1111/mpp.12565

**Published:** 2017-08-10

**Authors:** Ravi B. Anjanappa, Devang Mehta, Michal J. Okoniewski, Alicja Szabelska‐Berȩsewicz, Wilhelm Gruissem, Hervé Vanderschuren

**Affiliations:** ^1^ Department of Biology ETH Zurich 8092 Zurich Switzerland; ^2^ ID Scientific IT Services ETH Zurich 8092 Zurich Switzerland; ^3^ Functional Genomics Center Zurich 8057 Zurich Switzerland; ^4^ Department of Mathematical and Statistical Methods Poznan University of Life Sciences 60‐637 Poznan Poland; ^5^ AgroBioChem Department, Gembloux Agro‐Bio Tech University of Liège 5030 Gembloux Belgium

**Keywords:** callose, cassava, CBSV, ipomovirus, RDR1, RNA‐seq, salicylic acid, virus resistance

## Abstract

*Cassava brown streak virus* (CBSV) and *Ugandan cassava brown streak virus* (UCBSV) are responsible for significant cassava yield losses in eastern sub‐Saharan Africa. To study the possible mechanisms of plant resistance to CBSVs, we inoculated CBSV‐susceptible and CBSV‐resistant cassava varieties with a mixed infection of CBSVs using top‐cleft grafting. Transcriptome profiling of the two cassava varieties was performed at the earliest time point of full infection (28 days after grafting) in the susceptible scions. The expression of genes encoding proteins in RNA silencing, salicylic acid pathways and callose deposition was altered in the susceptible cassava variety, but transcriptional changes were limited in the resistant variety. In total, the expression of 585 genes was altered in the resistant variety and 1292 in the susceptible variety. Transcriptional changes led to the activation of β‐1,3‐glucanase enzymatic activity and a reduction in callose deposition in the susceptible cassava variety. Time course analysis also showed that CBSV replication in susceptible cassava induced a strong up‐regulation of *RDR1*, a gene previously reported to be a susceptibility factor in other potyvirus–host pathosystems. The differences in the transcriptional responses to CBSV infection indicated that susceptibility involves the restriction of callose deposition at plasmodesmata. Aniline blue staining of callose deposits also indicated that the resistant variety displays a moderate, but significant, increase in callose deposition at the plasmodesmata. Transcriptome data suggested that resistance does not involve typical antiviral defence responses (i.e. RNA silencing and salicylic acid). A meta‐analysis of the current RNA‐sequencing (RNA‐seq) dataset and selected potyvirus–host and virus–cassava RNA‐seq datasets revealed that the conservation of the host response across pathosystems is restricted to genes involved in developmental processes.

## Introduction

Cassava brown streak disease (CBSD) is one of the most damaging virus diseases of cassava in Africa, causing harvest losses of up to 70% in susceptible varieties (Hillocks *et al*., 2001; Legg *et al*., 2011). Ongoing CBSD outbreaks are threatening cassava production in sub‐Saharan Africa (Legg *et al*., 2011, 2014), and the recent spread of CBSD into central Africa (Bigirimana *et al*., 2011; Mulimbi *et al*., 2012) indicates that it could soon become a major constraint to cassava production in all sub‐Saharan cassava‐growing regions (Rey and Vanderschuren, 2017).

The characteristic symptoms of CBSD include leaf chlorosis and dry hard necrosis in roots, thus affecting both the quality and yield of the edible storage roots. CBSD is caused by two phylogenetically distinct potyvirus species: *Cassava brown streak virus* (CBSV) and *Ugandan cassava brown streak virus* (UCBSV) (Mbanzibwa *et al*., 2011), collectively referred to as CBSVs. CBSVs are positive single‐stranded RNA [(+)ssRNA] viruses belonging to the genus *Ipomovirus*, family *Potyviridae* (Mbanzibwa *et al*., 2009; Monger *et al*., 2001), and are transmitted by whiteflies (*Bemisia tabaci*) (Maruthi *et al*., 2005). CBSVs consist of an RNA genome of approximately 9 kb with a single open reading frame encoding a large polyprotein, which is co‐ and post‐translationally cleaved into 10 viral proteins (Mbanzibwa *et al*., 2009; Rey and Vanderschuren, 2017; Winter *et al*., 2010). CBSV and UCBSV differ in virulence in both controlled and field conditions, with CBSV generally accumulating to higher titres in most host genotypes (Kaweesi *et al*., 2014; Mohammed *et al*., 2012; Ogwok *et al*., 2015; Winter *et al*., 2010). Two recent field analyses of different cassava genotypes for the presence of CBSV and UCBSV found that both viruses occurred as mixed infections in the majority of tested genotypes (Kaweesi *et al*., 2014; Ogwok *et al*., 2015).

The release of a cassava reference genome (Prochnik *et al*., 2012) has opened up new opportunities to perform large‐scale characterization of the cassava transcriptome and proteome (Allie *et al*., 2014; Maruthi *et al*., 2014; Vanderschuren *et al*., 2014). Cassava varieties differing in resistance against cassava geminiviruses have been analysed recently in a time course experiment to identify pathways that are differentially regulated during *South African cassava mosaic virus* (SACMV) infection (Allie *et al*., 2014). Similarly, a late (1 year post‐infection) time point comparative transcriptome analysis has been reported of transcripts differentially expressed in a susceptible variety (Albert) and a variety (Kaleso) classified as moderately resistant, but displaying mild CBSD symptoms after CBSV infection (Maruthi *et al*., 2014). The late time point study found no change in expression of cassava homologues of known *R*‐gene analogues, nucleotide‐binding site‐leucine‐rich repeat (NBS‐LRR) genes or genes encoding proteins of the antiviral RNA silencing pathway. We have recently characterized KBH 2006/18 and KBH 2006/26, which are two resistant elite breeding lines with immunity to a mixed infection of CBSV and UCBSV (Anjanappa *et al*., 2016). Interestingly, KBH 2006/18 and KBH 2006/26 show no symptoms or hypersensitive response (HR) on infection, even at 16 weeks after graft inoculation (Anjanappa *et al*., 2016).

Here, we used two cassava varieties contrasting for CBSV resistance (i.e. 60444 and KBH 2006/18) to characterize the transcriptome response at an early time point of CBSV and UCBSV co‐infection. We report an in‐depth characterization of the pathways and genes altered in compatible and incompatible cassava–ipomovirus interactions and present biochemical evidence to explain the inhibition of virus intercellular movement in the resistant breeding line KBH 2006/18. These data provide new insights into the cassava–CBSV interaction and identify commonalities in plant responses to virus infection.

## Results

### CBSV inoculation induces greater transcriptome modulation in susceptible cassava

Following successful grafting of 60444 and KBH 2006/18 virus‐free scions onto rootstocks with a mixed infection of CBSV (TAZ‐DES‐01) and UCBSV (TAZ‐DES‐02) (CBSV infection in short), we performed a time course sampling of the scions at 16, 22 and 28 days after grafting (dag) (Fig. S1, see Supporting Information). We assessed virus titres in inoculated and mock scions at the selected time points. Virus infection reached homogeneous titre levels across the three biological replicates only at 28 dag in inoculated 60444 scions (Fig. S2, see Supporting Information). No virus was detected in leaves of KBH 2006/18 scions (Fig. S2), consistent with previous results (Anjanappa *et al*., 2016). Notably, we did not observe CBSD symptoms in any inoculated plant at 28 dag. Samples from 60444 and KBH 2006/18 scions at 28 dag were selected for transcriptome analysis.

Gene expression in 60444 and KBH 2006/18 from non‐inoculated and CBSV‐inoculated scions collected at 28 dag was analysed using Illumina HiSeq 2000 sequencing. Total RNA from the second emergent leaf immediately after the graft union was used for RNA‐sequencing (RNA‐seq) analysis, with three independent replicates per condition (Fig. S1) and per plant variety. A total of 1 254 619 280 reads with 100‐bp paired ends was generated from the 12 samples, ranging from 77 to 121 million reads per sample. On average, 93.4% of the total reads were successfully mapped to the cassava reference genome (*Manihot esculenta* AM560–2 v6.0). The reads not mapped to the reference genome were assembled *de novo* using Trinity (Grabherr *et al*., 2011). The size‐selected Trinity‐assembled contigs, referred to as *de novo* assembled transcripts (*dn*ATs), were subsequently blasted against the National Center for Biotechnology Information (NCBI) nucleotide collection to provide gene descriptions. The *dn*ATs were then used for differential gene expression analysis using STAR (Dobin *et al*., 2013). None of the *dn*ATs appeared to be differentially regulated in compatible and incompatible cassava–CBSV pathosystems (Table S2, see Supporting Information).

**Figure 1:**
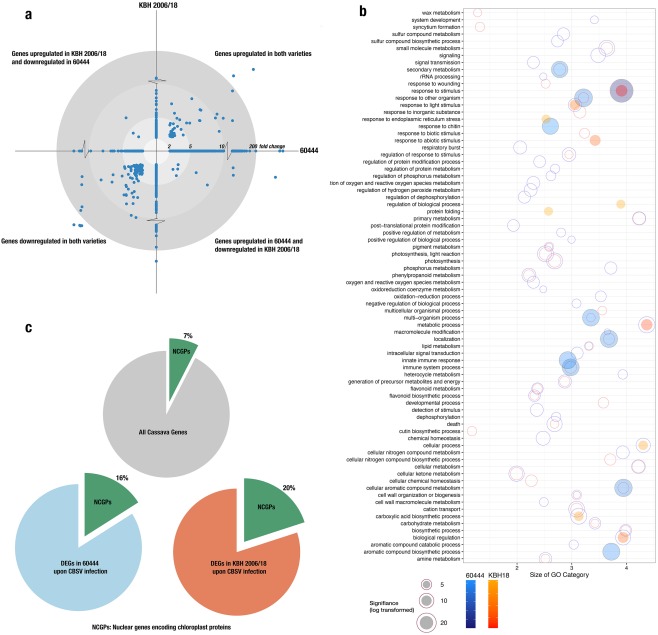
Gene regulation in susceptible (60444) and resistant (KBH 2006/18) cassava varieties at 28 days after grafting (dag) and mixed *Cassava brown streak virus* (CBSV) and *Ugandan cassava brown streak virus* (UCBSV) infection. (a) Fold change in differentially expressed genes (DEGs) [false discovery rate (FDR) < 0.05, fold change ≥ 2] in both varieties. Fold change in DEGs in 60444 form the *x*‐coordinate and in KBH 2006/18 form the *y*‐coordinate. (b) Functional characterization of DEGs based on gene ontology (GO) of biological processes. Significantly enriched GO categories in 60444 and KBH 2006/18 were first reduced based on semantic similarity (REVIGO; revigo.irb.hr) and then plotted. Blue circles represent GO categories enriched in 60444 and orange circles represent GO categories enriched in KBH 2006/18; overlapping blue and orange circles represent GO categories enriched in both 60444 and KBH 2006/18. Circles for the top 10 significantly enriched categories are shaded. (c) Proportion of nuclear genes encoding chloroplast proteins (NGCPs) differentially regulated after infection; 6% of the cassava genome consists of NGCPs, whereas 16% of 60444 and 20% of KBH 2006/18 DEGs after infection are NGCPs.

A total of 1292 genes in 60444 and 585 genes in KBH 2006/18 were differentially expressed at 28 dag, reflecting a higher number of modulated genes in the compatible host–virus interaction (Table S3, see Supporting Information). Over 68% of all differentially expressed genes (DEGs) were up‐regulated in variety 60444, whereas only 30% were up‐regulated in KBH 2006/18. A total of 158 DEGs were common between both varieties, and a major proportion (99 DEGs) of these were down‐regulated (Fig. 1a). Among the DEGs found in both plant varieties, only 12 genes were differently regulated (i.e. up‐regulated in 60444 and down‐regulated in KBH 2006/18, or vice versa) (Fig. 1a).

### Comparison and validation of RNA‐seq data

The RNA‐seq expression results of three significantly transcriptionally changed genes [*PLASMODESMATA LOCATED PROTEIN 1* (*PDLP1*), *CALRETICULIN 1B* (*CRT1B*), *HEAT SHOCK PROTEIN 17.6* (*HSP17.6*)] and one non‐significantly changed gene [*CALRETICULIN 3* (*CRT3*)] were confirmed using reverse transcription‐quantitative polymerase chain reaction (RT‐qPCR) on the same RNA samples as used for sequencing. Fold change values (comparing infected and non‐infected samples) for these RNAs were similar to those observed in the RNA‐seq analysis (Fig. S3, see Supporting Information). We used unmapped reads to *de novo* assemble the full‐length virus genomes of CBSV (TAZ‐DES‐01) and UCBSV (TAZ‐DES‐02). Full‐length virus genome contigs were employed for read counts to estimate virus abundance in each sample used for RNA‐seq (Fig. S4, see Supporting Information). Read counts detected for CBSV (TAZ‐DES‐01) and UCBSV (TAZ‐DES‐02) indicated that CBSV (TAZ‐DES‐01) was more prevalent than UCBSV (TAZ‐DES‐02) in two of the three inoculated 60444 scions. The read count analysis confirmed the absence of both CBSVs in the inoculated KBH 2006/18 scions (Fig. S4).

### Gene sets enriched in resistant and susceptible cassava

We performed Gene Set Enrichment Analysis using the PlantGSEA toolkit (Yi *et al*., 2013) to identify significantly enriched gene ontology (GO) categories and KEGG (Kyoto Encyclopedia of Genes and Genomes) pathways (Table S4, see Supporting Information). Significantly enriched GO categories (Biological Process) were then summarized and visualized based on semantic similarity using REVIGO (Supek *et al*., 2011) (Fig. 1b). As expected, the expression of genes related to stimulus response, immune system process and photosynthesis was significantly altered in both the susceptible and resistant plant varieties (Fig. 1b).

We also examined KEGG‐annotated pathways enriched after virus infection in both varieties by mapping DEGs onto enriched KEGG pathways using Pathview (Luo and Brouwer, 2013) (Table S4). In both resistant and susceptible varieties, genes in photosystems I and II, as well as the light‐harvesting complex, were down‐regulated. Pathways related to phenylalanine metabolism, phenylpropanoid biosynthesis and downstream products of phenylpropanoid biosynthesis were enriched in the infected 60444 scions, together with several genes involved in plant–pathogen interactions. In KBH 2006/18, the cyanoamino metabolism, starch and sucrose metabolism pathways were enriched. Interestingly, the phenylpropanoid pathway was also over‐represented in KBH 2006/18, but it showed an opposite response to the infected 60444 variety.

A recent transcriptome study has identified the down‐regulation of nuclear genes encoding chloroplast proteins (NGCPs) as a key response to pathogen‐associated molecular pattern (PAMP; specifically, bacterial pathogen) perception (Shi *et al*., 2015). Considering the over‐representation of chloroplast‐related genes (TAIR GO:0009507) in our datasets, we further investigated the relative abundance of NGCP transcripts regulated by CBSV infection. In 60444, 7% of all cassava NGCPs (193 of 2682 cassava NGCPs), which represent 15% of the total DEGs, were transcriptionally altered on infection. In KBH 2006/18, 19% of all DEGs are NGCPs (Fig. 1c). In both varieties, a majority of NGCPs were down‐regulated (Table S4), indicating that CBSV inoculation induces a chloroplastic response, as observed with other PAMPs in Arabidopsis (Göhre *et al*., 2012; de Torres Zabala *et al*., 2015; Zheng *et al*., 2012).

### CBSV infection modulates callose deposition at plasmodesmata

**Figure 2:**
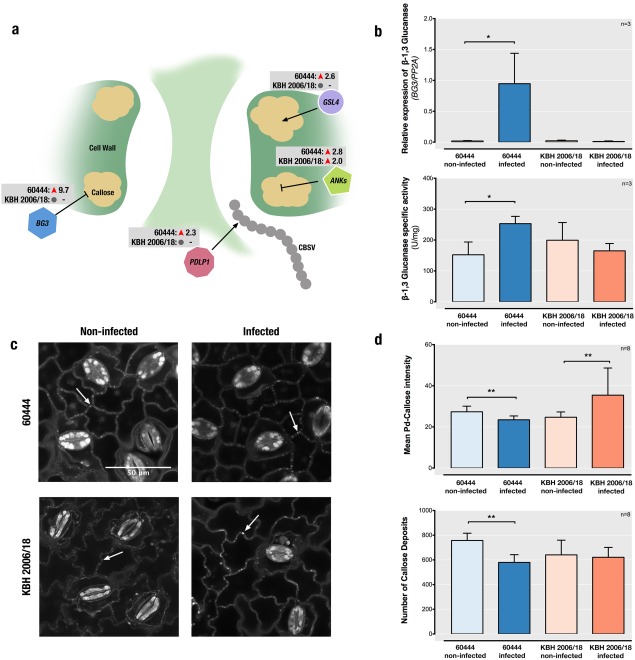
Regulation of callose deposition at plasmodesmata in susceptible (60444) and resistant (KBH 2006/18) cassava varieties after *Cassava brown streak virus* (CBSV) infection. (a) Schematic depiction of a plasmodesmata used by viruses for intercellular movement. Also shown are genes encoding key enzymes significantly regulated after infection (*BG3*, *GLUCANASE*, encoding a callose‐degrading β‐1,3‐glucanase; *GSL4*, *GLUCAN SYNTHASE‐LIKE 4*, encoding a callose synthesis enzyme; *PDLP1*, *PLASMODESMATA LOCATED PROTEIN 1*, encoding a protein involved in virus movement across the plasmodesmata and in callose synthesis; *ANK*s, *ANKYRIN REPEAT FAMILY PROTEINS*, encoding proteins that are negative regulators of callose deposition) with their fold changes based on RNA‐sequencing (RNA‐seq) analysis at 28 days after grafting (dag). (b) Top: reverse transcription‐quantitative polymerase chain reaction (RT‐qPCR) analysis of *BG3* mRNA at 60 dag. 60444 leaves were symptomatic for virus infection at this time point. Bottom: BG3 enzymatic activity also increases significantly after infection in 60444 and is unchanged in KBH 2006/18 at 60 dag. (c) Aniline blue staining for detection of callose deposits. Leaf samples from 60444 and KBH 2006/18 were stained and visualized using a confocal laser scanning microscope at 400× magnification. Callose deposits are visible as small bright dots along the cell walls (white arrowheads). (d) Top: quantification of callose deposits at plasmodesmata (Pd) in 60444 and KBH 2006/18 at 60 dag, observed after aniline blue staining and microscopy. Bottom: number of callose deposits found in microscopic images. Infection decreases the number of callose deposits in 60444. *P* values indicate statistical significance: * *P* < 0.05; **P < 0.01, unpaired *t*‐test.

In a previous study investigating the cassava–CBSV pathosystem, we found that KBH 2006/18 allows the transmission of CBSVs through the stem vasculature, whereas no virus can be detected in KBH 2006/18 leaves (Anjanappa *et al*., 2016). This suggests that the resistance of KBH 2006/18 to CBSVs involves at least restriction of intercellular virus movement from vascular tissues to mesophyll cells. Here, we found that transcript levels of genes involved in virus movement and callose deposition at plasmodesmata were altered in infected 60444, but not infected KBH 2006/18, leaves. The transcript level of *β‐1,3‐GLUCANASE* (*BG3*), encoding an enzyme which degrades callose at the plasmodesmata (Iglesias and Meins Jr, 2000), was 9.7‐fold up‐regulated in 60444 (Fig. 2a). ANKYRIN REPEAT FAMILY PROTEINS (ANKs) allow viral movement through plasmodesmata by promoting callose degradation (Ueki *et al*., 2010). We also found an up‐regulation of two different cassava *ANK*s: Manes.11G035500 (3.8‐fold) in 60444 and Manes.05G056700 (2.03‐fold) in KBH 2006/18. The expression of *PDLP1*, which is a positive regulator of virus cell‐to‐cell movement, was up‐regulated in infected 60444. PDLP1 binds to viral movement proteins and aids in the formation of viral movement tubules at the plasmodesmata (Amari *et al*., 2010), thereby facilitating virus cell‐to‐cell movement. In contrast, the expression of a possible negative regulator of plasmodesmata permeability, *GLUCAN SYNTHASE LIKE 4* (*GSL4*) (Maeda *et al*., 2014), was also up‐regulated by 2.6‐fold in 60444 (Fig. 2a).

We further characterized *BG3* expression levels at 60 dag to follow the regulation of this gene in infected and symptomatic 60444 leaves. Up‐regulation of *BG3* expression was attenuated at 60 dag as CBSV infection progressed. Consistently, no significant change in *BG3* expression was observed in KBH 2006/18 leaves at 60 dag (Fig. 2b). The higher *BG3* transcript levels resulted in a 38% increase in BG3 enzymatic activity in infected 60444 leaves (Fig. 2b).

In order to understand the functional implications of the simultaneous increase in expression of positive (*PDLP1*, *GSL4*) and negative (*BG3*, *ANK*s) regulators of callose deposition on CBSV infection, we stained callose in leaf tissue from 60444 and KBH 2006/18 at 60 dag (Fig. 2c). This revealed a significant decrease in plasmodesmata‐associated callose deposition in epidermal cells of infected 60444 leaves, and a strong increase in the amount of plasmodesmata‐associated callose deposition in KBH 2006/18 leaves.

### Salicylic acid (SA) defence and lignin biosynthesis pathways are altered in response to CBSVs

**Figure 3:**
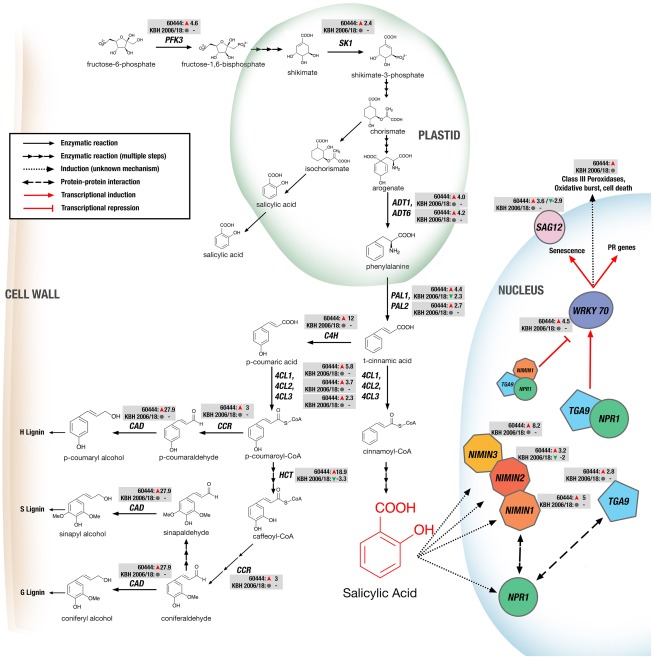
Regulation of genes encoding proteins of the salicylic acid synthesis and signalling and lignin synthesis pathways in cassava after virus infection. Significantly regulated genes are depicted together with their mRNA fold change based on RNA‐sequencing (RNA‐seq) analysis in susceptible (60444) and resistant (KBH 2006/18) cassava varieties after infection. *PFK3*, *PHOSPHOFRUCTOKINASE 3*; *SK1*, *SHIKIMATE KINASE 1*; *ADT1*, *ADT6*, *AROGENATE DEHYDRATASE 1 and 6*; *PAL1*, *PAL2*, *PHENYLALANINE AMMONIA‐LYASE 1 and 2*; *C4H*, *CINNAMATE‐4‐HYDROXYLASE*; *4CL1*, *4CL2*, *4CL3*, *4‐COUMARATE‐CoA LIGASE 1, 2 and 3*; *HCT*, *HYDROXYCINNAMOYL‐CoA SHIKIMATE/QUINATE HYDROXYCINNAMOYL TRANSFERASE*; *CCR*, *CINNAMOYL CoA REDUCTASE 1*; *CAD*, *CINNAMOYL ALCOHOL DEHYDROGENASE*; *NPR1*, *NON‐EXPRESSOR OF PR GENES 1*; *NIMIN1*, *NIMIN2*, *NIMIN3*, *NIM1‐INTERACTING*; *TGA9*, *TGACG MOTIF‐BINDING PROTEIN 9*; *WRKY70*, *WRKY DNA‐BINDING PROTEIN 70*; *SAG12*, *SENESCENCE‐ASSOCIATED GENE 12*.

Overall, we found that, in the susceptible cassava variety 60444, defence pathways were more extensively altered during CBSV infection than in the resistant variety KBH 2006/18. The transcript levels of several genes encoding the enzymes involved in lignin and SA biosynthesis were up‐regulated in the compatible cassava–CBSV interaction and unchanged or down‐regulated in the incompatible interaction (Fig. 3). Both SA and lignin biosynthetic pathways follow the phenylpropanoid pathway, which was enriched in our initial KEGG pathway gene set analysis (Table S4).

SA biosynthesis in plants begins in the chloroplast with the shikimate pathway for the synthesis of aromatic amino acids, which, in turn, is fed by fructose‐1,6‐bisphosphate (Fig. 3). Although fructose‐1,6‐bisphosphate is probably not a limiting precursor for SA biosynthesis, we did observe a 4.6‐fold up‐regulation of *PHOSPHOFRUCTOKINASE 3*, a gene involved in the conversion of fructose‐6‐phosphate to fructose‐1,6‐bisphosphate, in the susceptible variety. We also observed a 2.4‐fold up‐regulation of *SHIKIMATE KINASE 3*, responsible for the conversion of shikimate to shikimate‐3‐phosphate. Previous genetic studies in Arabidopsis and *Nicotiana benthamiana* (reviewed by Chen *et al*., 2009) have indicated that pathogen‐induced SA synthesis is catalysed by ISOCHORISMATE SYNTHASE (ICS) to convert chorismate to isochorismate, which is then used to produce SA by an unknown mechanism. An alternative pathway for SA biosynthesis from chorismate involves its conversion to arogenate, which is the substrate of AROGENATE DEHYDRATASE (ADT) to produce phenylalanine. *ADT1* and *ADT6* were up‐regulated in 60444 after CBSV infection, suggesting that virus‐induced SA synthesis in cassava, unlike in *N. benthamiana* (Catinot *et al*., 2008), might proceed through the phenylalanine pathway rather than via isochorismate. PHENYLALANINE AMMONIA‐LYASE (PAL), which catalyses the conversion of phenylalanine to cinnamic acid, is a key enzyme in the synthesis of both lignin and SA. Indeed, quadruple *pal* mutants with only 10% PAL activity produced about 50% less SA and were more susceptible to biotic stress (Huang *et al*., 2010). We found that *PAL1* (Manes.10G047500) and *PAL2* (Manes.07G098700) were 4.4‐fold and 2.7‐fold up‐regulated, respectively, in 60444. In contrast, *PAL1* was down‐regulated 2.3‐fold in KBH 2006/18. Cinnamic acid is the substrate at the branch point of the SA and lignin biosynthesis pathways. Ligation of cinnamic acid to coenzyme A by 4‐COUMARATE:CoA LIGASE (4CL) leads to SA biosynthesis via both peroxisomal β‐oxidative and cytosolic non‐oxidative pathways. The conversion of cinnamic acid, first to *p*‐coumaric acid by CINNAMATE‐4‐HYDROXYLASE (C4H), and then ligation of coenzyme A to *p*‐coumaric acid by 4CL, produces *p*‐coumaroyl‐CoA, which is a phenylpropanoid pathway precursor (Widhalm and Dudareva, 2015). In our study, *4CL1* (Manes.14G151400, Manes.04G095300), *4CL2* (Manes.11G071800), *4CL3* (Manes.09G127000.1) and *C4H* (Manes.02G227200) were all up‐regulated in CBSV‐infected 60444 (Fig. 3).

SA is a key regulator of plant defence responses and has been implicated in various mechanisms resulting in the HR and systemic acquired resistance (SAR) (reviewed in Vlot *et al*., 2009). One such mechanism is the NON‐EXPRESSOR OF PR GENES 1 (NPR1)‐dependent response (Vlot *et al*., 2009), which involves the SA‐induced activation and nuclear localization of NPR1 to activate the transcription of genes for pathogenesis‐related (PR) proteins, including BG3. NPR1 interacts with various members of the TGA (TGACG MOTIF‐BINDING PROTEIN 9) family of transcription factors, which together activate the expression of WKRY transcription factors (Vlot *et al*., 2009). We found that *TGA9* (Manes.04G004100) expression was up‐regulated by 2.8‐fold in CBSV‐infected 60444, together with the up‐regulation of two cassava *WRKY70* genes (Manes.07G142400 by 4.5‐fold and Manes.10G002200 by 2.5‐fold). In Arabidopsis, WRKY70 is an SA‐induced transcription factor that activates the expression of several PR genes, including *SENESCENCE‐ASSOCIATED GENE*s (*SAG*s) encoding a peroxidase and senescence‐associated proteins (*SAG12*, *SAG21*, *SAG24*, *SAG29*) (Li *et al*., 2004; Ülker *et al*., 2007). In CBSV‐infected 60444, *SAG12* (Manes.16G038400) was up‐regulated 3.6‐fold (Fig. 3, Table S3). Similarly, the genes encoding 10 of 14 Class III peroxidases (Prxs, or PR‐9 subfamily), which are involved in SA‐induced pathogen defence responses (Almagro *et al*., 2009), were up‐regulated (Fig. 3).

Lignins are polymers of aromatic compounds and integral components of the plant cell wall. They play a role in defence against microbial and fungal pathogens (Miedes *et al*., 2014; Vanholme *et al*., 2010). Lignin synthesis is often induced in plant immune responses (Malinovsky *et al*., 2014). We found several genes encoding enzymes involved in the production of phenylpropanoid intermediates and their conversion to the three monolignols that were up‐regulated after CBSV infection in 60444 (Fig. 3, Table S3).

### The antiviral RNA silencing pathway is up‐regulated in the compatible cassava–CBSVs interaction

**Figure 4:**
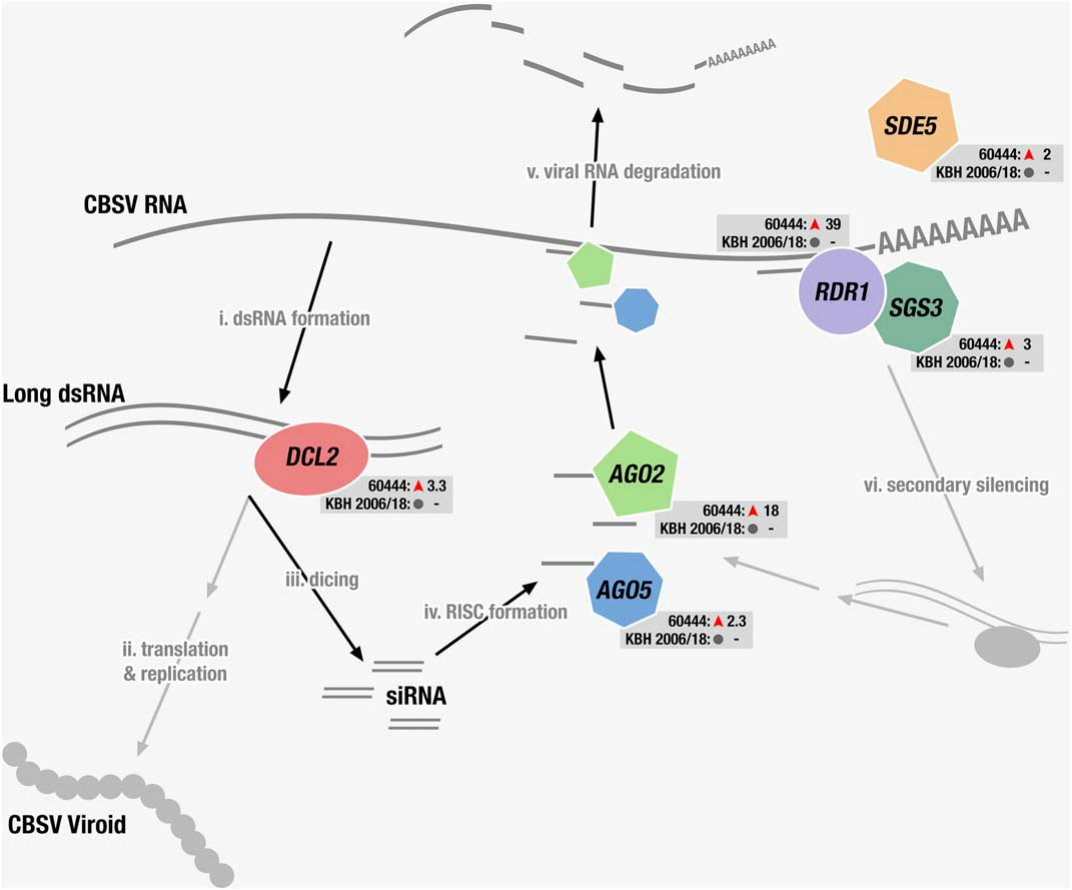
Schematic depiction of the antiviral RNA silencing pathway with differentially expressed genes (DEGs) encoding proteins that function in antiviral defence. Genes significantly regulated in variety 60444 after inoculation are shown together with their mRNA fold change based on RNA‐sequencing (RNA‐seq) analysis. These genes were not regulated in variety KBH 18/2006. *DCL2*, *DICER‐LIKE 2*; *AGO5*, *ARGONAUTE 5*; *AGO2*, *ARGONAUTE 2*; *RDR1*, *RNA‐DEPENDENT RNA POLYMERASE 1*; *SGS3*, *SUPPRESSOR OF GENE SILENCING 3*; *SDE5*, *SILENCING DEFECTIVE 5*; RISC, RNA‐induced silencing complex.

RNA silencing is a major defence mechanism against plant viruses (Wang *et al*., 2012), especially against RNA viruses, because their genomes and replicative forms can be targeted directly by RNA‐induced silencing complexes (RISCs) and DICER‐LIKE (DCL) proteins (Waterhouse and Fusaro, 2006). RNA silencing is thought to begin with the synthesis and formation of long double‐stranded RNA (dsRNA) molecules, which are substrates of DCL proteins in plants (Fig. 4) (Pumplin and Voinnet, 2013). In Arabidopsis, four DCL proteins are involved in RNA silencing, of which DCL2 and DCL4 have overlapping functions in antiviral defence (Garcia‐Ruiz *et al*., 2010). However, DCL4 alone is sufficient for antiviral RNA silencing (Garcia‐Ruiz *et al*., 2010). DCL2 appears to be required for the production of secondary small interfering RNAs (siRNAs) (Parent *et al*., 2015). We found that the expression of three cassava *DCL2* genes was increased in CBSV‐infected 60444 (Manes.12G002700 by 2.4‐fold, Manes.12G002800 by 3.3‐fold and Manes.12G003000 by 3.3‐fold).

ARGONAUTE (AGO) proteins are essential constituents of RISCs, which are involved in the sequence‐specific silencing of genes by RNA degradation, inhibition of translation and epigenetic modifications. Of the 10 AGO proteins known in plants, AGO2 has a broad and probably specific role in antiviral silencing (Carbonell *et al*., 2012; Jaubert *et al*., 2011; Scholthof *et al*., 2011). Expression of the cassava *AGO2* gene (Manes.07G023100) was 17.5‐fold up‐regulated in CBSV‐infected 60444 scions. A gene expressing AGO5 (Manes.04G011400), which is another less well‐characterized AGO protein, was also up‐regulated 2.3‐fold. The virus‐induced increase in expression of *AGO2* and *AGO5* in cassava is similar to that in *N. benthamiana*, in which *Potato virus X* infection increases *AGO5* expression and AGO5 acts synergistically with AGO2 to suppress virus infection to a greater degree than the other AGO proteins (Brosseau and Moffett, 2015).

RNA‐DEPENDENT RNA POLYMERASE 1 (RDR1) is an important component of the post‐transcriptional gene silencing (PTGS) immune response to virus infections and is involved in the defence of various classes of viruses, including potyviruses (Garcia‐Ruiz *et al*., 2010; Lee *et al*., 2016; Rakhshandehroo *et al*., 2009; Wang *et al*., 2010). RDRs catalyse the synthesis of the antisense strand in the conversion of viral RNA to a long dsRNA template that can be processed by DCLs. PTGS is often initiated by the binding of siRNAs to a target and the recruitment of RDRs, which then results in the production of further long dsRNA templates for the production of secondary siRNAs. This mechanism, also known as transitivity or silencing amplification, is largely facilitated by RDR6, and possibly also RDR1 (Wang *et al*., 2010). We found that the expression of *RDR1* (Manes.17G084800) was highly up‐regulated (38.5‐fold) in CBSV‐infected 60444 scions and not altered in KBH 2006/18 scions (Fig. 4). The steep up‐regulation of *RDR1* was concomitant with virus replication at 28 dag (Fig. S5, see Supporting Information). Previous studies have shown that RDR1 can act as a silencing suppressor contributing to virus susceptibility in a potyvirus–*N. benthamiana* pathosystem (Ying *et al*., 2010). RDR1 also contributes to the production of virus‐activated siRNAs (vasiRNAs) involved in the regulation of host genes during virus infection (Cao *et al*., 2014). In total, 369 genes have been identified previously as potential RDR1 targets, and the *RDR1*‐dependent reduction in transcription of the *PHOTOSYSTEM II LIGHT HARVESTING COMPLEX B1.3* (*LHCB1.3*) gene in response to potyvirus infection has been validated in Arabidopsis. We consistently observed a 3.4‐fold down‐regulation of *LHCB1.3* (Manes.17G066700) in CBSV‐infected 60444 scions. However, of the 26 putative RDR1 target genes [of the 369 genes identified by Cao *et al*. (2014)] changed in our study, only 13 were down‐regulated (Table S6, see Supporting Information).

The expression of the PTGS‐related genes *SILENCING DEFECTIVE 5* (*SDE5*) and *SUPPRESSOR OF GENE SILENCING 3* (*SGS3*) was also significantly up‐regulated in CBSV‐infected 60444 (Fig. 3, Table S3), but not changed in CBSV‐infected KBH 2006/18. *AGO2* and *RDR1*, which are both up‐regulated in CBSV‐infected 60444, are also induced by SA signalling pathways (Hunter *et al*., 2013; Jovel *et al*., 2011; Lee *et al*., 2016; Lewsey *et al*., 2010), which is consistent with the up‐regulation of several cassava genes involved in SA synthesis and signalling pathways after CBSV infection. The expression of genes relating to antiviral RNA silencing was not significantly changed in the resistant variety.

### Similar transcript regulation in selected host–potyvirus and cassava–virus pathosystems

We performed a comparative analysis of the DEGs detected above with other RNA‐seq studies of host–virus pathosystems to identify similarities and differences in gene expression. The studies selected involved naturally occurring crop–virus interactions with detectable host responses to potyvirus infections and comparable RNA‐seq datasets (late‐stage cassava–CBSV infection, Maruthi *et al*., 2014; early‐stage peach and late‐stage apricot infection with *Plum pox virus*, Rubio *et al*., 2015a, 2015b). We also included an RNA‐seq study of cassava infected with SACMV, a ssDNA geminivirus pathosystem (Allie *et al*., 2014), to compare the response of cassava to two different virus types.

**Figure 5:**
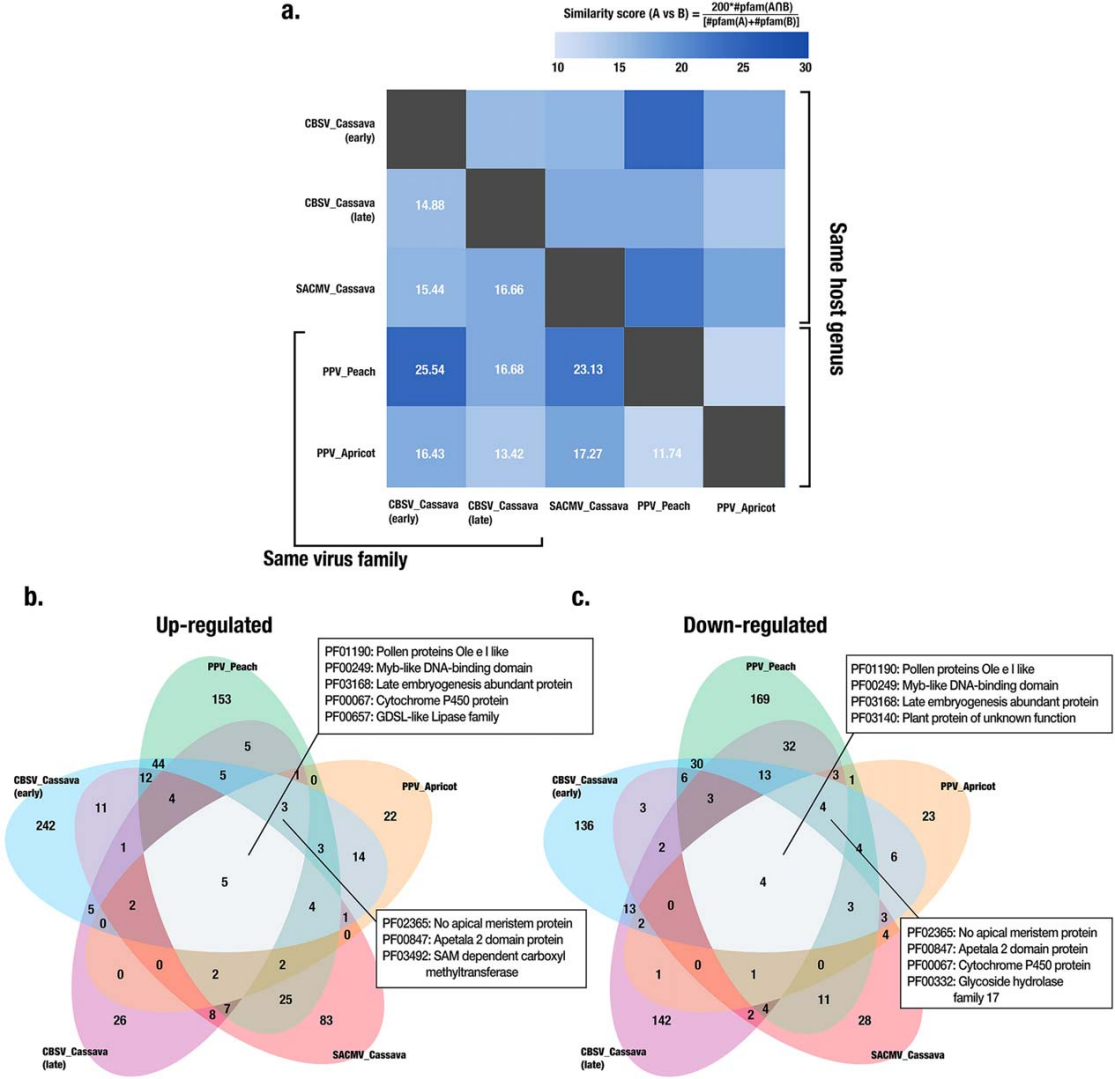
Comparison of transcriptome changes in different virus–host systems. We compared our RNA‐sequencing (RNA‐seq) of *Cassava brown streak virus* (CBSV)‐infected cassava with three other potyvirus–plant transcriptome studies [late‐stage cassava–CBSV infection, Maruthi *et al*., 2014; early‐stage peach and late‐stage apricot infection with *Plum pox virus* (PPV), Rubio *et al*., 2015a, 2015b]. In addition, we included an RNA‐seq study of cassava infected with *South African cassava mosaic virus* (SACMV), a single‐stranded DNA (ssDNA) geminivirus pathosystem (Allie *et al*., 2014), to compare the response of cassava to two different virus types. (a) Pairwise similarity matrix of numbers of differentially expressed genes (DEGs) in each pathosystem, based on comparison of Pfam identifiers associated with each gene. A pairwise percentage similarity score was computed by multiplying the DEGs found in common in both pathosystems by 200 and then dividing this number by the sum of the DEGs in both pathosystems that were compared. (b, c) Venn diagrams were constructed using the Pfam identifiers associated with DEGs from all five studies. The figure shows specific Pfam identifiers common to all five studies, as well as those common to all potyvirus studies. (b) Up‐regulated DEGs. (c) Down‐regulated DEGs. [Correction added on 12 January 2018 after first online publication: the formula in figure 5(a) has been revised. The figure caption has also been revised to reflect the changes in the formula.]

We first filtered DEGs from all datasets according to our differential expression criteria (fold change > 2; FDR < 0.01) and used Pfam identifiers (Finn *et al*., 2015) matching each DEG as a common term to compare gene expression between cassava and the two *Prunus* species. This allowed us to construct a similarity matrix based on a pairwise similarity percentage score, where 100% denotes complete similarity (Fig. 5a, grey boxes). The greatest pairwise similarity (25.54%) was found between peach and cassava RNA‐seq datasets obtained within 2 months post‐infection. Interestingly, we found more common Pfam identifiers between our early time point cassava–CBSV dataset and the early time point cassava–SACMV dataset, rather than between the two cassava–CBSV datasets. In addition, the two cassava–CBSV datasets and the two *Prunus* datasets had a lower degree of similarity than most other pairs, indicating a strong temporal component to the modulation of host gene expression after virus infection. Our meta‐analysis did not reveal a strong dependence of host gene expression changes on either RNA or DNA virus or host genus.

We next compared up‐ and down‐regulated DEGs separately across the five datasets (Fig. 5b,c; Table S5, see Supporting Information) and found five Pfam identifiers in common for up‐regulated and four for down‐regulated DEGs. Of these, three Pfams that contained genes in both the up‐ and down‐regulated datasets in all pathosystems included PF01190 (pollen protein Ole e I‐like), PF00249 (Myb‐like DNA‐binding domain) and PF03168 (late embryogenesis‐abundant protein). Although PF01190 represents a largely uncharacterized protein family, the other two identifiers comprise stress response genes. Genes belonging to PF00067 were up‐regulated in all pathosystems and this Pfam comprises the cytochrome P450 protein family, which includes members involved in the synthesis of plant defence compounds. Genes encoding members of the GDSL‐like lipase family (PF00657) were also up‐regulated in all pathosystems and are required for systemic ethylene‐mediated resistance (Kim *et al*., 2013; Kwon *et al*., 2009).

In addition to the Pfams common to all RNA‐seq datasets, genes in another three Pfams were found to be up‐regulated and four down‐regulated in all potyvirus infection studies (Fig. 5b,c). Of these, genes belonging to PF02365 (NO APICAL MERISTEM protein) and PF00847 (APETALA 2 domain protein) were found in both the up‐ and down‐regulated datasets.

## Discussion

We have shown that the transcriptional response of cassava to CBSV infection is significantly stronger in the susceptible 60444 variety than in the resistant KBH 2006/18 variety. This is similar to results reported previously for extreme virus resistance in potato (Goyer *et al*., 2015). To ensure that our dataset represented the earliest time point of complete virus infection, we first measured virus titres in the inoculated scions at three time points until we found consistent infection in all 60444 plants (Fig. S2). This allowed us to analyse changes in gene expression prior to symptom development, and thus early in the infection process. Although graft inoculation may delay infection until the graft junction is fully established, we have demonstrated previously consistent infection using the top‐grafting method (Anjanappa *et al*., 2016; Moreno *et al*., 2011; Vanderschuren *et al*., 2012). Graft virus transmission using field‐infected rootstocks or scions was necessary because no infectious CBSV clones are available (Anjanappa *et al*., 2016; Mohammed *et al*., 2012; Vanderschuren *et al*., 2012; Wagaba *et al*., 2013). Although the lack of detectable virus titres in KBH 2006/18 indicates the absence of viral replication in the resistant cultivar, we observed a transcriptional response to graft inoculation. It is currently unknown whether this is a result of the local recognition of viral RNAs and/or proteins, which were mobilized in the vascular tissues after the graft was established, or of an extended systemic response transmitted by the CBSV‐susceptible rootstock to the CBSV‐resistant scion.

Based on the DEGs in the susceptible variety 60444, we found that CBSV infection increases the expression of genes encoding SA synthesis enzymes, which probably increases SA levels to activate SA‐regulated genes involved in antiviral responses. Our data suggest that, in CBSV‐infected cassava, SA is synthesized via the phenylalanine rather than isochorismate pathway. In addition, we found that genes encoding proteins of the DCL2/AGO5/AGO2‐mediated antiviral silencing response were up‐regulated in CBSV‐infected 60444, but not KBH 2006/18. The silencing response may be amplified as indicated by the strong up‐regulation of the gene encoding RDR1, but it is also possible that RDR1 assists in the repression of RDR6‐mediated silencing amplification, as previously reported in *N. benthamiana* inoculated with *Plum pox virus* (Ying *et al*., 2010).

The susceptible 60444 and resistant KBH 2006/18 varieties differed strongly in callose deposition at plasmodesmata during CBSV infection. Although DEGs associated with virus movement through plasmodesmata were found only in CBSV‐infected 60444, callose deposition at plasmodesmata was reduced in 60444 and increased in KBH 2006/18, which could only be partially explained by the detected DEGs. Nevertheless, the results are consistent with the limitation of CBSV movement in KBH 2006/18 from stems into leaves (Anjanappa *et al*., 2016). An efficacious CBSV infection of the susceptible 60444 variety thus involves a virus‐induced reduction in callose deposition at the plasmodesmata that is absent in the resistant KBH 2006/18 variety.

The meta‐analysis of our RNA‐seq dataset and other plant–virus RNA‐seq datasets revealed a low level of similarity in host transcriptome responses to different virus types and at different time points of infection. A previous transcriptome‐based meta‐analysis of plant–virus interactions compared transcriptional responses in Arabidopsis after infection with different virus species (Rodrigo *et al*., 2012). The results indicated a correlation between host transcriptome response and virus phylogeny, with closely related viruses generating transcript regulation of similar host genes. As our analysis did not involve a model plant, we managed differences in genome annotations in different species using Pfam identifiers. Although Pfam identifiers reduce resolution because they represent protein families instead of specific genes, this approach does not require the identification of exact gene orthologues across multiple species. Thus, this allows the comparison of transcriptome regulation between different viruses and plant species. Overall, we found only a small number of common Pfam identifiers, even between different hosts of the same genus or between viruses of the same family. However, based on the numbers of common Pfam identifiers, there was a strong temporal impact on gene regulation after virus infection, indicating that earlier transcript level changes are perhaps most diagnostic to infection by different viruses. Pfam identifiers that were found in common across the selected pathosystems did not relate to classical defence pathways, but rather to protein families implicated in developmental processes. A previous review of expression profile studies also found commonalities within developmental genes across various compatible pathosystems (Whitham *et al*., 2006). The common regulation of developmental genes might describe similarities in symptom development or downstream signalling processes, rather than primary immune responses, across different host–virus interactions.

Our study suggests that the analysis of early time points during virus–host interactions captures the emerging impact of virus replication on host gene expression. For example, our RNA‐seq dataset from scion leaves at 28 dag revealed a broad immune response (including SA and RNA silencing pathways) in the susceptible cassava 60444 variety which was not found in susceptible cassava plants after a long‐term infection (Maruthi *et al*., 2014). Our meta‐analysis of different RNA‐seq datasets also showed significant differences in transcriptome responses at different times after infection. The absence of a detectable immune response in the resistant KBH 2006/18 variety suggests that even earlier time points after infection should be analysed, preferably using *Agrobacterium* with infectious CBSV clones or infections of protoplasts followed by transcriptomics or proteomics. The Pfam‐based meta‐analysis also identified interesting protein families, such as meristem identity proteins [no apical meristem (NAM) and Apetala 2], whose expression was altered in different host–potyvirus interactions and at different time points after infection. Understanding the functions of these proteins in host–virus interactions might reveal conserved developmental responses to virus infections across different taxa. Our Pfam‐based comparison methodology also makes a larger scale comparison of crop RNA‐seq studies possible, with the aim of developing a more comprehensive understanding of conserved responses of non‐model plants to different biotic and abiotic stresses.

## Experimental Procedures

### Plant material and virus inoculation

The elite CBSV‐resistant cassava breeding variety KBH 2006/18 was obtained from the International Institute of Tropical Agriculture (IITA), Dar es Salaam, Tanzania, and the susceptible variety 60444 from the ETH Zurich (Zurich, Switzerland) cassava germplasm collection. Cassava plants were grown under glasshouse conditions (27 °C, 16 h light, 60% humidity). Infected 60444 rootstocks with mixed infection of the CBSV isolate TAZ‐DES‐01 (KF878104) and the UCBSV isolate TAZ‐DES‐02 (KF878103) were employed to inoculate KBH 2006/18 and 60444 plants using a previously described top‐cleft grafting method (Anjanappa *et al*., 2016; Moreno *et al*., 2011). KBH 2006/18 and 60444 plants were also top‐cleft grafted onto virus‐free 60444 rootstocks as mock controls. Individual leaves (the second leaf from the point of graft union) were collected from scions of CBSV‐ and mock‐infected plants at 28 and 60 dag (Fig. S1).

### RT‐qPCR

RNA was extracted from cassava leaves using a modified cetyltrimethylammonium bromide (CTAB) RNA extraction protocol (Moreno *et al*., 2011). DNaseI‐treated total RNA (1 μg) was reverse transcribed using a RevertAid First Strand cDNA Synthesis Kit (Thermo‐Fisher, Waltham, Massachusetts, USA) according to the manufacturer's instructions. RT‐qPCR was performed with SYBR Green dye (Thermo‐Fisher) using a 7500 Fast Real Time PCR System (Applied Biosystems, Waltham, Massachusetts, USA). Three independent biological replicates (i.e. leaf samples from three individual plants) were analysed for each time point. The relative expression level of individual genes in each sample was calculated as described previously (Moreno *et al*., 2011; Vanderschuren *et al*., 2012). The primers used to validate DEGs and to quantify virus titres are listed in Table S1 (see Supporting Information).

### Illumina RNA‐seq and data analysis

Library preparation and sequencing were performed as follows. Five micrograms of total RNA extract were treated with DNase according to the manufacturer's instructions (Qiagen, RNase‐free DNase Kit), and subsequently column purified using an RNeasy Plant Mini Kit (Qiagen, Hilden, Germany). RNA quality was assessed using a Qubit® (1.0) Fluorometer (Thermo‐Fisher) as well as a Bioanalyzer 2100 (Agilent, Santa Clara, California, USA). Samples with an RNA integrity number (RIN) above six were used for sequencing. One microgram of total RNA was polyA enriched and cDNA libraries were synthesized using a TruSeq RNA Sample Prep Kit v2 (Illumina, San Diego, California, USA). Cluster generation was performed using 10 pm of pooled normalized libraries on the cBOT with a TruSeq PE Cluster Kit v3‐cBot‐HS (Illumina), and, subsequently Illumina HiSeq 2000 sequencing was performed to generate the reads.

### Sequence assembly, mapping to the cassava genome and identification of DEGs

The raw read files were trimmed using the trim sequence tool in CLC Genomics Workbench v8.5, and the settings are declared in Notes S1 (see Supporting Information). Unique trimmed reads were mapped to the *M. esculenta* reference genome AM560–2 v6.0 available on Phytozome (www.phytozome.jgi.doe.gov) using the transcriptomics toolbox in CLC Genomics Workbench v8.5. Empirical analysis of differentially expressed reads was performed using default parameters to identify DEGs between control non‐infected and CBSVs‐infected scions. DEGs were then filtered based on an FDR‐corrected *P* value of <0.01 and a fold change of ≥2. DEGs were annotated in CLC Workbench using the Phytozome GFF3 annotation file (www.phytozome.jgi.doe.gov).

### Identification of *dn*ATs

Read mapping of trimmed reads was performed using the transcriptomics toolbox in CLC Genomics Workbench v8.5 with the settings described in Notes S1, but with ‘Maximum number of hits for a read = 10’, allowing for more stringent detection of unmapped reads. Unmapped reads for each cassava variety were pooled and assembled using Trinity (Grabherr *et al*., 2011) with default settings. Assembled contigs were then size filtered and contigs greater than 1000 nucleotides were classified as *dn*ATs. Differential expression of *dn*ATs between infected and mock‐infected samples was determined using STAR (Dobin *et al*., 2013).

### Determination of full‐length virus genomes from RNA‐seq analysis

Unmapped reads (described previously) were assembled into contigs using the CLC Genomics Workbench *de novo* assembly tool. The assembled contigs were blasted against published CBSV (HG965221.1) and UCBSV (HG965222.1) genomes to identify full‐length virus genomes. Two full‐length genomes corresponding to CBSV (TAZ‐DES‐01; KF878104) and UCBSV (TAZ‐DES‐02; KF878103) were obtained as single assembled contigs. The two full‐length viral genomes were used to map reads from each individual sample (Fig. S1) using the CLC Genomics Workbench read mapping to contigs tool in order to estimate virus read counts per sample and read count distribution across the virus genomes.

### β‐1,3‐Glucanase enzymatic assay

Leaf samples from three independent cassava scions were collected, and soluble proteins were extracted from 100 mg of leaf powder in 300 µL of extraction buffer [20 mm HEPES, pH 8.0, 5 mm MgCl_2_ and 1 tablet/50 mL buffer of ethylenediaminetetraacetic acid (EDTA)‐free protease inhibitor] and quantified using the bicinchoninic acid assay (BCA) assay. The amount of glucose released by the activity of β‐1,3‐glucanase in a known amount of soluble proteins in the presence of the substrate laminarin (*Laminaria digitata*, Sigma‐Aldrich, St. Louis, Missouri, USA) was then measured spectrophotometrically at 540 nm (Ramada *et al*., 2010). β‐1,3‐Glucanase specific activity was subsequently calculated, with 1 unit (U) of activity representing the amount of enzyme required to produce 1 µmol of reducing sugar per minute.

### Quantification of plasmodesmata‐associated callose

Leaf sections from uninfected and infected scions were stained overnight with aniline blue (Merck, Darmstadt, Germany) using a previously described method (Guenoune‐Gelbart *et al*., 2008). Stained leaf samples were then examined using a Zeiss (Oberkochen, Germany) LSM 780 laser confocal microscope with a 40× water immersion objective. Callose accumulation was detected at an excitation of 405 nm and emission between 413 and 563 nm was recorded. Callose quantification and deposit counting were performed as described by Zavaliev and Epel (2015) for eight replicate images per plant variety and treatment condition.

### Pfam‐based meta‐analysis

For Pfam‐based meta‐analysis, RNA‐seq DEG data were downloaded from published reports (Allie *et al*., 2014; Rubio *et al*., 2015a, 2015b; Maruthi *et al*., 2014) and filtered using an absolute fold change of ≥2. Pfam annotations for genomes of each species were obtained from Phytozome annotation files (www.phytozome.jgi.doe.gov). Commonly regulated Pfams were determined by constructing a five‐set Venn diagram, and the resulting Pfam overlaps between pairs of sets were used to calculate a pairwise similarity matrix.

## Supporting information

Additional Supporting Information may be found in the online version of this article at the publisher's website:

**Fig. S1** Experimental scheme for the study. (a) Schematic representation of the workflow for the RNA‐sequencing (RNA‐seq) study. (b) Workflow for callose quantification, enzymatic assay and expression analysis for β‐1,3‐glucanase.
**Fig. S2** *Cassava brown streak virus* (CBSV) quantification at three different time points after grafting. Reverse transcription‐quantitative polymerase chain reaction (RT‐qPCR) quantification of virus titre [log_2_ fold change relative to reference gene *Manihot esculenta protein phosphatase 2A* (*MePP2A*)] from individual leaves from three independent biological replicates at three different time points after grafting.
**Fig. S3** Validation of RNA‐sequencing (RNA‐seq) data by reverse transcription‐quantitative polymerase chain reaction (RT‐qPCR). RT‐qPCR for four genes was performed on the same samples as used for RNA‐seq analysis. *MePP2A*, *Manihot esculenta protein phosphatase 2A*.
**Fig. S4** Virus read counting from unmapped RNA‐sequencing (RNA‐seq) reads. Unmapped reads from each sample sent for RNA‐seq were mapped to the two *de novo* assembled virus genomes. (a) Read distribution across virus genomes for 60444 samples. (b) Read counts for all samples in the compatible (60444) and incompatible (KBH 2006/18) virus–host interaction.
**Fig. S5** Reverse transcription‐quantitative polymerase chain reaction (RT‐qPCR)‐based RNA‐DEPENDENT RNA POLYMERASE 1 (RDR1) transcript expression across the three time points. dag, days after grafting; *MePP2A*, *Manihot esculenta protein phosphatase 2A*.
**Table S1** Primers used in this study.

**Table S2** Significantly differentially expressed genes from RNA‐seq analysis.

**Table S3** Identification and differential expression of *de novo* assembled transcripts.

**Table S4** Gene set analysis results.

**Table S5** Comparison of multiple virus–host RNA‐sequencing (RNA‐seq) studies.

**Table S6** Differentially expressed genes (DEGs) in the study that match putative RNA‐DEPENDENT RNA POLYMERASE 1 (RDR1) targets based on Cao *et al*. (2014).

**Notes S1** Settings used for transcriptomics analysis and visualisation.
