# Supplementary material for: Molecular insights into Cassava brown streak virus susceptibility and resistance by profiling of the early host response
Source: Mol Plant Pathol. 2017 Aug 10;19(2):476–89. doi: 10.1111/mpp.12565 (PMC6638049; doi:10.1111/mpp.12565)
Supplement: Supplementary file 1 — Fig. S1 Experimental scheme for the study. (a) Schematic representation of the workflow for the RNA‐sequencing (RNA‐seq) study. (b) Workflow for callose quantification, enzymatic assay and expression analysis for β‐1,3‐glucanase. Fig. S2 Cassava brown streak virus (CBSV) quantification at three different time points after grafting. Reverse transcription‐quantitative polymerase chain reaction (RT‐qPCR) quantification of virus titre [log2 fold change relative to reference gene Manihot esculenta protein phosphatase 2A (MePP2A)] from individual leaves from three independent biological replicates at three different time points after grafting. Fig. S3 Validation of RNA‐sequencing (RNA‐seq) data by reverse transcription‐quantitative polymerase chain reaction (RT‐qPCR). RT‐qPCR for four genes was performed on the same samples as used for RNA‐seq analysis. MePP2A, Manihot esculenta protein phosphatase 2A. Fig. S4 Virus read counting from unmapped RNA‐sequencing (RNA‐seq) reads. Unmapped reads from each sample sent for RNA‐seq were mapped to the two de novo assembled virus genomes. (a) Read distribution across virus genomes for 60444 samples. (b) Read counts for all samples in the compatible (60444) and incompatible (KBH 2006/18) virus–host interaction. Fig. S5 Reverse transcription‐quantitative polymerase chain reaction (RT‐qPCR)‐based RNA‐DEPENDENT RNA POLYMERASE 1 (RDR1) transcript expression across the three time points. dag, days after grafting; MePP2A, Manihot esculenta protein phosphatase 2A. Table S1 Primers used in this study. [file MPP-19-476-s001.docx]

## Supporting Information

Article title: Early transcriptome analysis of the brown streak virus–cassava pathosystem provides molecular insights into virus susceptibility and resistance

Authors: Ravi B. Anjanappa*, Devang Mehta*, Alicja Szabelska, Michal Okoniewski, Wilhelm Gruissem and Hervé Vanderschuren

The following Supporting Information is available for this article:

**Fig. S1** **Experimental scheme for the study**

1. Schematic representation of the workflow for RNA-seq study.
2. **
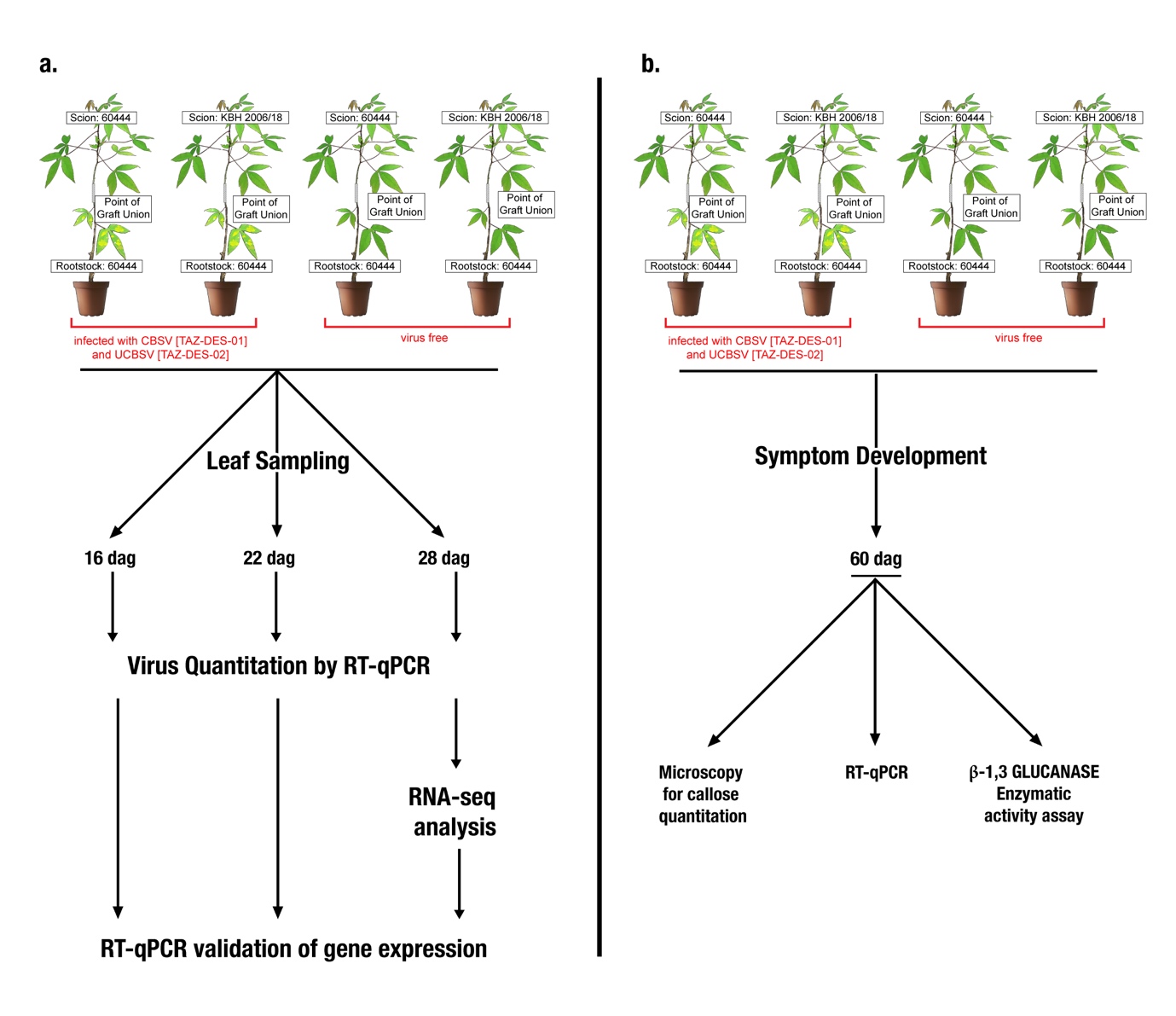
**Workflow for callose quantitation, enzymatic assay and expression analysis for β 1,3 Glucanase.

**Fig. S2** **CBSV quantitation at 3 different time points after grafting**

RT-qPCR quantitation of virus titre (log2 fold change relative to reference gene mePP2A) from individual leaves from 3 independent biological replicates at three different time points after grafting.

**
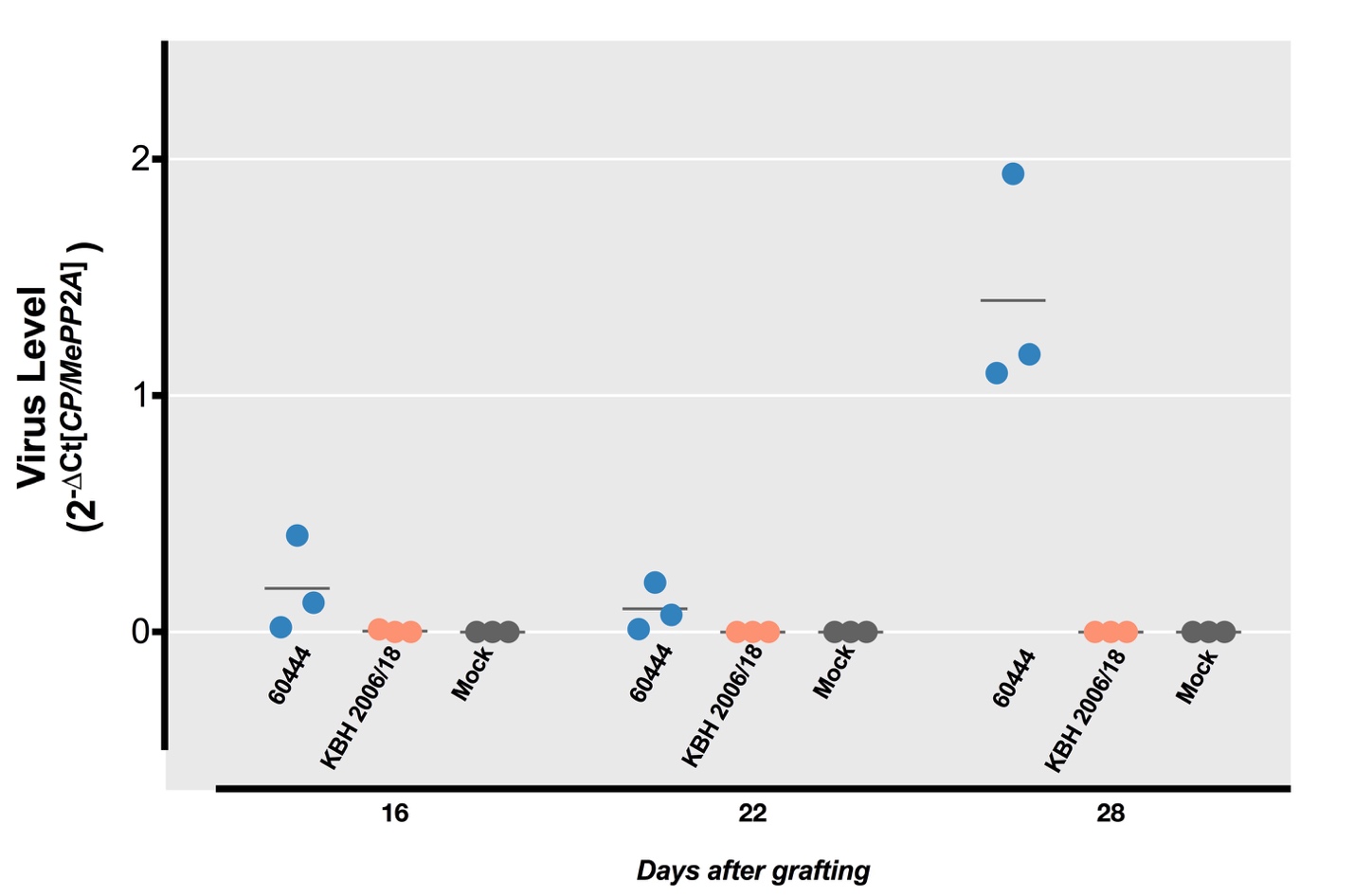
**

**Fig S3 Validation of RNA-seq data by RT-qPCR**

RT-qPCR for four genes was performed on the same samples that were used for RNA-seq analysis.


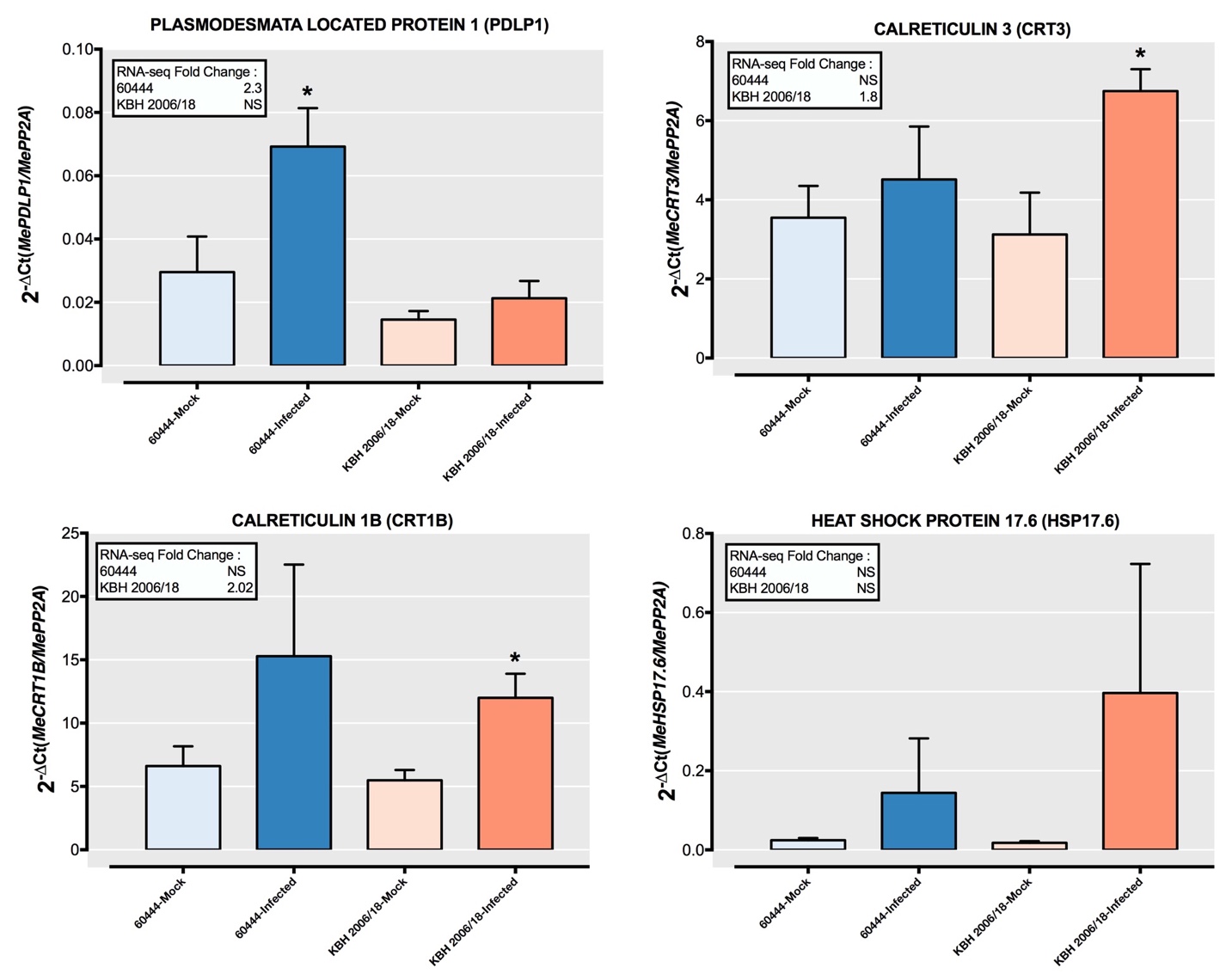


**Fig. S4 Virus read counting from unmapped RNA-seq reads**

Unmapped reads from each sample sent for RNA-seq were mapped to the two *de novo* assembled virus genomes. **a.** Read distribution across virus genomes for 60444 samples. **b.** Read counts for all samples in the compatible (60444) and incompatible (KBH 2006/18) virus-host interaction.

**
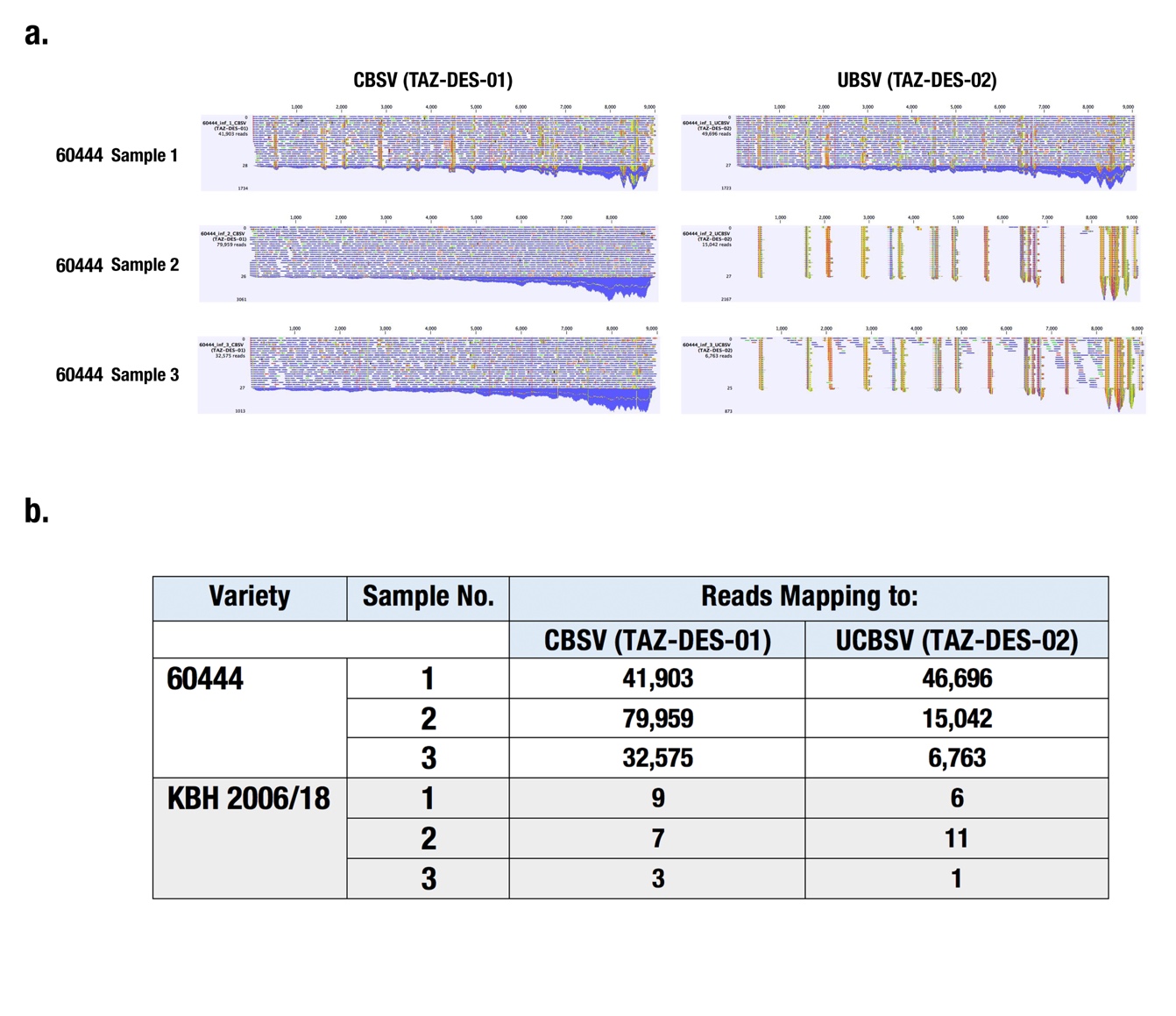
**

**Fig. S5** **RT-qPCR based RDR1 transcript expression across the three time points**

**22 DAG**

**16 DAG**

**28 DAG**

**
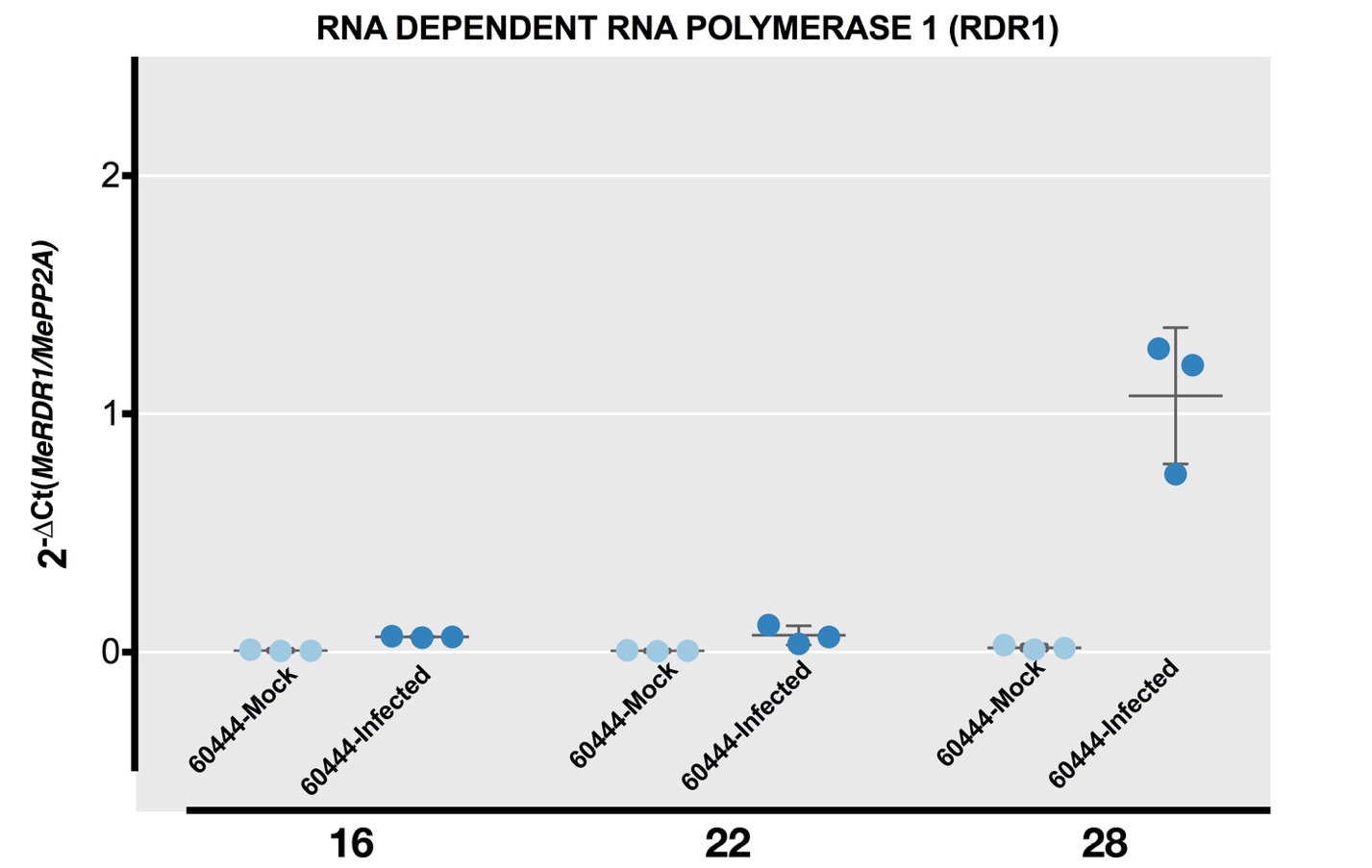
**

**Table S1 Primers used in the study**

| Gene Id | Gene | Primer Name | Forward | Reverse |
| --- | --- | --- | --- | --- |
|  | CBSV *COAT PROTEIN* | CBSV-CP3 | GAAGTTGAGAATTGGGCCATC | CAGCTCTCCACGATTTCTCATT |
| Manes.09G039900 | *PROTEIN PHOSPHATASE 2A-4* | MePP2A | TGCAAGGCTCACACTTTCATC | CTGAGCGTAAAGCAGGGAAG |
| Manes.17G084800 | *RNA-DEPENDENT RNA POLYMERASE 1* | MeRDRP1 | CAAATCAAACAACACCAGCCTAGA | GCCTCCTCTGCCTGTAATGG |
| Manes.13G040200 | *PLASMODESMATA-LOCATED PROTEIN 1* | MePDLP1 | AAAGGTGAAGCAGTCAATTCCAA | CAAAGCAGCACACAGCAAGAA |
| Manes.07G031600 | *TOBAMOVIRUS MULTIPLICATION 1* | MeTOM1 | ACACAGGCATGCATCTGGATAT | GAACCGGCTGAGCATGATAAA |
| Manes.06G107800 | *CDC48* | CDC48 | TGACAGAAATGGATGGCATGAC | GAAAGCGGGAGGCTTCATCT |
| Manes.08G112000 | *CALRETICULIN 1B* | MeCRT1b | AAGGGTCCATGGAAGCAAAA | CACCTGCCACAACTCAATACCA |
| Manes.13G003400 | *CALRETICULIN 3* | MeCRT3 | CATGGAGGCCCAAGAGAATC | CAGCCTTCACCTGCCAACT |
| Manes.16G083600 | *17.6 KDA CLASS II HEAT SHOCK PROTEIN* | MeHSP17.6 | GGAAAGAAGGGTTGGCAAGTT | TCGATGGTCTTGGGCTTTTT |
| Manes.08G054400 | *Β-1,3-GLUCANASE 3* | MeBG3 F | GCGGAGGAGCTTTGGAGATT | CAGTGGGTTTTCCAGGCTTCT |

**Table S2 Significantly differentially expressed genes from RNA-seq analysis**

List of significant (FDR <0.01) and differentially expressed genes (infected vs mock) with fold change >2. Annotations displayed are from Phytozome cassava genome V6.

**Table S3 Identification and differential expression of De novo assembled transcripts**

List of denovo assembled transcripts with a contig length >1000 and associated log2 fold change values and statistics.

**Table S4 Gene set analysis results**

Significantly enriched gene ontology categories (BP and MF), significantly enriched KEGG pathways and list of nuclear encoded chloroplast genes (NECGs) for 60444 and KBH 2006/18.

**Table S5 Comparison of multiple virus-host RNA-seq studies**

Datasets from four RNA-seq studies. Common up and down-regulated PFAMs across the fpathosystems compared.

**Table S6 DEGs in the study that match putative RDR1 targets based on Cao *et al.,* 2014.**
